# Gene expression variation to predict 10-year survival in lymph-node-negative breast cancer

**DOI:** 10.1186/1471-2407-8-254

**Published:** 2008-09-08

**Authors:** Elin Karlsson, Ulla Delle, Anna Danielsson, Björn Olsson, Frida Abel, Per Karlsson, Khalil Helou

**Affiliations:** 1 Department of Oncology, Institute of Clinical Sciences, Blå stråket 2, University of Gothenburg, SE-413 45, Göteborg, Sweden; 2 School of Life Sciences, University College of Skövde, Box 408, SE-541 28, Skövde, Sweden; 3 Genomics Core Facility, Medicinaregatan 5A, Box 413, SE-405 30, Göteborg Sweden; 4 Oncology section, Department of Oncology, Sahlgrenska University Hospital, Blå, stråket 2, University of Gothenburg, SE-413 45, Göteborg, Sweden

## Abstract

**Background:**

It is of great significance to find better markers to correctly distinguish between high-risk and low-risk breast cancer patients since the majority of breast cancer cases are at present being overtreated.

**Methods:**

46 tumours from node-negative breast cancer patients were studied with gene expression microarrays. A t-test was carried out in order to find a set of genes where the expression might predict clinical outcome. Two classifiers were used for evaluation of the gene lists, a correlation-based classifier and a Voting Features Interval (VFI) classifier. We then evaluated the predictive accuracy of this expression signature on tumour sets from two similar studies on lymph-node negative patients. They had both developed gene expression signatures superior to current methods in classifying node-negative breast tumours. These two signatures were also tested on our material.

**Results:**

A list of 51 genes whose expression profiles could predict clinical outcome with high accuracy in our material (96% or 89% accuracy in cross-validation, depending on type of classifier) was developed. When tested on two independent data sets, the expression signature based on the 51 identified genes had good predictive qualities in one of the data sets (74% accuracy), whereas their predictive value on the other data set were poor, presumably due to the fact that only 23 of the 51 genes were found in that material. We also found that previously developed expression signatures could predict clinical outcome well to moderately well in our material (72% and 61%, respectively).

**Conclusion:**

The list of 51 genes derived in this study might have potential for clinical utility as a prognostic gene set, and may include candidate genes of potential relevance for clinical outcome in breast cancer. According to the predictions by this expression signature, 30 of the 46 patients may have benefited from different adjuvant treatment than they recieved.

**Trial registration:**

The research on these tumours was approved by the Medical Faculty Research Ethics Committee (Medicinska fakultetens forskningsetikkommitté, Göteborg, Sweden (S164-02)).

## Background

Since the prevalence of breast cancer among women is very high (one out of eight American women is affected in their life-time [1]) the economical burden for the treatment is considerable, as well as the suffering it causes. After surgery the majority of breast cancer cases are at the present over-treated since adequate diagnostic markers are not currently available. Therefore it is important to find better markers to correctly distinguish between the high-risk patients that need additional treatment and the low-risk patients where further treatment after surgery will have no positive effect and might actually harm the patient. At present, the most relevant marker in breast cancer diagnostics is the lymph-node status of the patient. Although lymph-node negative breast cancer patients have a better survival rate than patients with metastasis positive lymph nodes, around 20% will succumb to their cancer in less than 15 years [2]. In this study, gene expression microarrays were used to find a set of genes whose expression profiles can predict clinical outcome in lymph-node negative breast cancer patients. We also wanted to evaluate our results on data sets derived from similar studies done previously on tumours from lymph-node negative patients [3,4].

## Methods

### Tumour material

Breast tumours from 46 patients consisting of two groups were used, 23 tumours from 10-year survivors and 23 tumours from patients that died within 10 years from diagnosis. The tumours were collected between 1990 and 1998 in the region Västra Götaland in Sweden. Clinical information about the tumours used in the study is compiled in table 1 and in more detail in additional data file 1; Clinical data of each patient. The median follow-up time among the survivors was 11.5 years (mean 11.8 years, range 10.4 to 15.3 years). In order to achieve a ten year follow-up and mitigate age-related diseases, samples were preferably selected from patients with a mean age and tumour size of 50 years old and 27 mm, respectively. Thus, age and size are not random and a multivariate analysis is therefore not possible. In addition, patients that died in intercurrent disease were excluded from the study. The research on these tumours was approved by the Medical Faculty Research Ethics Committee (Medicinska fakultetens forskningsetikkommitté, Göteborg, Sweden (S164-02)).

**Table 1:** Compilation of clinical data of the breast cancer patients used in the study.

	10-year survivors	deceased	**Total**
**Mean age**	**48**	**53**	**50**
**Surgery**			
breast preserving	11	11	**22**
mastectomy	12	12	**24**
**total**	**23**	**23**	**46**
			
**Histology**			
ductal	19	14	**33**
lobular	1	2	**3**
mucinous	2	0	**2**
medullary	0	1	**1**
tubular	0	1	**1**
comedocarcinoma	0	1	**1**
adenocarcinoma	0	1	**1**
not available	1	3	**4**
**total**	**23**	**23**	**46**
			
**Receptor Status**			
estrogen positive	12	11	**23**
progesterone positive	11	5	**16**
unavailable	1	0	**1**
			
**Ploidi**			
diploid	7	3	**10**
aneuploid	13	13	**26**
polyploid	2	2	**4**
not available	1	5	**6**
**total**	**23**	**23**	**46**
			
**S-phase**			
< 12%	14	7	**21**
>= 12%	3	8	**11**
not available	6	8	**14**
**total**	**23**	**23**	**46**

Representative imprints from each of the frozen tumours were evaluated for the ratio of cancer/normal cells. The imprints were air dried and stained with May-Grünwald-Giemsa (Chemicon, Temecula, CA, USA). The presence of at least 50% cancer cells was required for the specimen to be included in this study.

### Expression profiling

Microarrays were produced at the Swegene DNA Microarray Resource Center, Department of Oncology, Lund University, Sweden [5]. Human array-ready 70-mer oligonucleotide libraries Version 3.0, comprising approximately 35 000 unique probes, were obtained from Operon (Operon Biotechnologies, Germany). Probes were dissolved in Corning Universal Spotting solution (Corning, Acton, MA, USA) at a concentration of 24 μM and printed on aminosilane coated glass slides (UltraGAPS, Corning) using a MicroGrid2 robot (BioRobotics, Cambridgeshire, UK) equipped with MicroSpot 10 K pins (BioRobotics). Following printing, the arrays were left in a desiccator to dry for 48 hours, and then UV cross-linked (800 mJ/cm2).

Frozen tumours were homogenized in TRIzol Reagent (Invitrogen, Carlsbad, CA, USA) using a Micro-Dismembrator S (B. Braun Biotech International, Melsungen, Germany). From the cell-suspension total RNA was extracted using RNeasy mini kit (Qiagen, Valencia, CA, USA) according to the manufacturer's protocol. The quality of the RNA was evaluated with the Agilent 2100 BioAnalyser (Agilent Technologies, Palo Alto, CA, USA). Specimens where the 28S/18S ratio was lower than 1.0 or the RNA integrity number (RIN) -value [6] was lower than 6.7 were excluded from the study. For each sample, probes labelled with Cy3-dCTP (Amersham Biosciences, Buckinghamshire, UK) were synthesized from 5 μg of the total tumour RNA and reference labelled with Cy5-dCTP (Amersham Biosciences) was synthesized from 5 μg of commercial reference RNA (Universal Human Reference RNA, Stratagene, La Jolla, CA, USA) by reverse transcription. The probes were purified using ChipShot™ labelling cleanup system (Promega, Madison, WI, USA). The hybridizations were carried out using Pronto! Micro Array reagent systems (Corning Inc., Corning, NY, USA). For each sample, labelled tumour cDNA and reference cDNA were co-precipitated and hybridised to the microarray slide. The microarray slides were scanned with Agilent microarray scanner G2565AA (Agilent Technologies) and image analysis was performed using the Genepix 6.0.0.45 software (Axon Instruments, Union City, CA, USA). The expression data is available online at the Gene Expression Omnibus repository [7,8], accession number GSE12071.

### Data analysis

The first steps of data analysis were performed in BioArray Software Environment (BASE) [9,10]. The intensities of the spots were calculated by subtracting median background intensity from median spot intensity. Flagged spots and spots for which background adjusted intensities were < 0 or > 65 000 were excluded from further analysis. Spots with intensities below 20 in both channels were also excluded and intensities below 20 in one channel were set to 20 to compensate for extreme quotients. These steps reduced the number of genes from 34 659 to 31 564. Fluorescence ratios were calculated as intensity tumour/intensity reference. Each array was separately normalised using pin-based Lowess normalization with 12 blocks in each group. Reporters that were missing in more than 20% of the arrays were removed from the analysis. This reduced the dataset from 31 564 to 16 023 genes remaining for the statistical analysis. To identify differently expressed genes a t-test was applied and a list of 55 reporters with *p *< 0.001 was derived. All 55 genes also had *p *< 0.01 in a Mann-Whitney test and 37 of the genes showed Mann-Whitney *p*-values < 0.001. In order to avoid elimination of true positive genes, a low *p*-value threshold was used instead of false discovery rate (FDR) adjustment. Consequently, it is expected that 16 of the 55 genes might be false positives (29%), but the evaluation indicates that there is nevertheless a significant value of the expression signature as predictor of clinical outcome in breast cancer. Four of the 55 genes were not found in Entrez gene [11] and could not be connected to any known gene by a BLAST search with 100% correspondence. They were therefore excluded.

The 51 remaining significant genes (table 2) were used for hierarchical clustering of the samples using Euclidian distance and average linkage (UPGMA) in the PermutMatrix software [12]. We also tested a correlation-based classifier similar to the one van't Veer *et al*. used [3]. For the correlation classifier the log-ratio values were normalised to the scale [0–1] for each gene. Accuracy was calculated as the number of correctly classified samples divided by the total number of samples and the Negative Predictive Value (NPV) was calculated as the number of samples correctly classified as "good prognosis" divided by the total number of samples classified as "good prognosis". The threshold for the "good prognosis group" was set to 0.3 since it showed the highest NPV and a high accuracy. Kaplan-Meier curves were calculated for the "good prognosis group" and the "bad prognosis group".

**Table 2:** The genes that significantly differed between survivors and deceased patients.

**A**					
**Gene Symbol**	**Accesion number**	**Gene Name**	**Expression deceased vs 10-year survivors**	**Gene Ontology, Function**	**van't Veer/Wang**

**ADA**	NM_000022	adenosine deaminase	Higher	adenosine deaminase activity, hydrolase activity	1/1
**BCAT1**	NM_005504	branched chain aminotransferase 1, cytosolic	Higher	branched-chain-amino-acid transaminase activity, catalytic activity, transferase activity	1/1
**C9orf164**	NM_006378	chromosome 9 open reading frame 164	Higher		0/0
**CCDC99**	NM_017785	coiled-coil domain containing 99	Higher		1/0
**CCNB1IP1**	NM_182849	cyclin B1 interacting protein 1	Higher	ligase activity, metal ion binding	1/1
**COMMD9**	NM_014186	COMM domain containing 9	Higher		0/1
**CPS1**	NM_001875	carbamoyl-phosphate synthetase 1, mitochondrial	Higher	ATP binding, carbamoyl-phosphate synthase (ammonia) activity, ligase activity, nucleotide binding, protein binding	1/2
**E2F2**	NM_004091	E2F transcription factor 2	Higher	protein binding, transcription factor activity	1/1
**F2**	NM_000506	coagulation factor II (thrombin)	Higher	calcium ion binding, receptor binding, thrombin activity, thrombin activity	1/1
**GGH**	NM_003878	gamma-glutamyl hydrolase (conjugase, folylpolygammaglutamyl hydrolase)	Higher	exopeptidase activity, hydrolase activity	1/1
**GIT2**	NM_014776	G protein-coupled receptor kinase interactor 2	Higher	GTPase activator activity, metal ion binding	2/2
**GNG10**	NM_004125	guanine nucleotide binding protein (G protein), gamma 10	Higher	GTPase activity, heat shock protein binding, signal transducer activity	1/0
**HPS5**	NM_007216	Hermansky-Pudlak syndrome 5	Higher	protein binding	1/1
**KCNAB2**	NM_003636	potassium voltage-gated channel, shaker-related subfamily, beta member 2	Higher	ion channel activity, oxidoreductase activity, potassium channel regulator activity, potassium ion binding	1/0
**MGC13057**	NM_032321	hypothetical protein MGC13057	Higher		0/0
**MRTO4**	NM_016183	mRNA turnover 4 homolog (S. cerevisiae)	Higher		0/1
**MTERF**	NM_006980	mitochondrial transcription termination factor	Higher	DNA binding, double-stranded DNA binding, transcription termination factor activity	1/1
**MYO1G**	NM_033054	myosin IG	Higher	ATP binding, motor activity	0/0
**NPM3**	NM_006993	nucleophosmin/nucleoplasmin, 3	Higher	nucleic acid binding	1/0
**PIR**	NM_003662	pirin (iron-binding nuclear protein)	Higher	metal ion binding, transcription cofactor activity	1/1
**PLEKHM2**	XM_290944	pleckstrin homology domain containing, family M (with RUN domain) member 2	Higher	oxidoreductase activity	0/0
**PRPS1L1**	NM_175886	phosphoribosyl pyrophosphate synthetase 1-like 1	Higher	kinase activity, lipoate-protein ligase B activity, magnesium ion binding, ribose phosphate diphosphokinase activity, transferase activity	0/1
**RAB23**	NM_183227	RAB23, member RAS oncogene family	Higher	GTP binding, nucleotide binding	1/1
**RCN1**	NM_002901	reticulocalbin 1, EF-hand calcium binding domain	Higher	calcium ion binding	0/0
**RP5-1077B9.4**	NM_021933	invasion inhibitory protein 45	Higher		1/0
**SALL4**	NM_020436	sal-like 4 (Drosophila)	Higher	DNA binding, metal ion binding, nucleic acid binding, protein binding	0/0
**SERPINB9**	NM_004155	serpin peptidase inhibitor, clade B (ovalbumin), member 9	Higher	protein binding, serine-type endopeptidase inhibitor activity	1/2
**SLC35B4**	NM_032826	solute carrier family 35, member B4	Higher	UDP-N-acetylglucosamine transporter activity, UDP-xylose transporter activity, nucleotide-sugar transporter activity, sugar porter activity	0/0
**SPATA5**	NM_145207	spermatogenesis associated 5	Higher	ATP binding, nucleoside-triphosphatase activity, nucleotide binding	0/0
**TM4SF5**	NM_003963	transmembrane 4 L six family member 5	Higher		1/0
**TRPV2**	NM_016113	transient receptor potential cation channel, subfamily V, member 2	Higher	calcium ion binding, ion channel activity	2/0
					
**B**					

**Gene Symbol**	**Accesion number**	**Gene Name**	**Expression deceased vs 10-year survivors**	**Gene Ontology, Function**	**van't Veer/Wang**

**ARFIP1**	NM_001025595	ADP-ribosylation factor interacting protein 1 (arfaptin 1)	Lower	identical protein binding	1/0
**ARSD**	NM_001669	arylsulfatase D	Lower	arylsulfatase activity, calcium ion binding, hydrolase activity	2/2
**C1orf43**	NM_015449	chromosome 1 open reading frame 43	Lower		1/0
**C4orf26**	NM_178497	chromosome 4 open reading frame 26	Lower		0/0
**CCDC24**	NM_152499	coiled-coil domain containing 24	Lower		0/0
**CUL7**	NM_014780	cullin 7	Lower	protein binding	1/2
**EDEM3**	NM_025191	ER degradation enhancer, mannosidase alpha-like 3	Lower	calcium ion binding, mannosyl-oligosaccharide 1,2-alpha-mannosidase activity, peptidase activity	0/0
**FAAH**	NM_001441	fatty acid amide hydrolase	Lower	amidase activity, hydrolase activity, receptor binding	2/1
**FXYD3**	NM_005971	FXYD domain containing ion transport regulator 3	Lower	chloride ion binding, ion channel activity	1/0
**GOLT1A**	NM_198447	golgi transport 1 homolog A (S. cerevisiae)	Lower		1/0
**NEIL1**	NM_024608	nei endonuclease VIII-like 1 (E. coli)	Lower	DNA N-glycosylase activity, damaged DNA binding, hydrolase activity, acting on glycosyl bonds, lyase activity, oxidized purine base lesion DNA N-glycosylase activity, zinc ion binding	1/0
**OR7E91P**	NR_002185	olfactory receptor, family 7, subfamily E, member 91 pseudogene	Lower	receptor activity	0/0
**PHLDA3**	NM_012396	pleckstrin homology-like domain, family A, member 3	Lower		1/0
**PLEKHA6**	NM_014935	pleckstrin homology domain containing, family A member 6	Lower		1/1
**RORC**	NM_005060	RAR-related orphan receptor C	Lower	metal ion binding, sequence-specific DNA binding, steroid hormone receptor activity, transcription factor activity	1/1
**TAF5L**	NM_014409	TAF5-like RNA polymerase II, p300/CBP-associated factor (PCAF)-associated factor, 65 kDa	Lower	transcription factor activity	1/1
**TMEM63A**	NM_014698	transmembrane protein 63A	Lower		1/4
**ZNF497**	NM_198458	zinc finger protein 497	Lower	metal ion binding, nucleic acid binding	0/0
**ZNF691**	NM_015911	zinc finger protein 691	Lower	metal ion binding, nucleic acid binding	0/0
**ZNF692**	NM_017865	zinc finger protein 692	Lower	metal ion binding, nucleic acid binding	1/0

A, Biological function of the genes with significantly higher expression in the tumours from deceased patients compared to the tumours from 10-year survivors.

B, Biological function of the genes with significantly lower expression in the tumours from deceased patients compared to the tumours from 10-year survivors.

Gene Symbol, Gene Name and Gene Ontology information was captured from Entrez Gene. The last column shows the number of replicates in the suydies by van't Veer *et al*. and Wang *et al*., respectively.

In addition, forty-three classification methods available in the Weka software [13] were tested for their ability to classify the tumours. Each method was evaluated using leave-one-out cross-validation (46 testing and training cycles per method, using 45 samples for training and the remaining sample for testing in each run, and with each test-classification being considered correct if the left-out sample is classified in the correct survival group). The VFI classifier [14] turned out to achieve best results (additional data file 2. Classification results for our own data).

Data sets from other investigations with node-negative breast cancer tumours [3,4] were used for evaluation of our list of genes in order to validate our findings. We used the VFI classifier (since it performed best on our material) and the correlation-based classifier (since it was used by van't Veer *et al*. [3]) to evaluate the predictive performance of our genes in the other sets of tumours. Furthermore, these two studies generated two different gene sets containing 70 and 76 genes, respectively, with the ability to predict metastasis within five years [3,4]. We tested the prediction value of the expression signatures from these gene lists on our tumours by the VFI classification method and correlation-based classification (using the same threshold for our data as earlier, i.e. 0.3).

Another analysis was performed in BASE using only reporters present in the entire assay-set A t-test analysis was carried out and resulted in 94 reporters that differed significantly between the tumours from 10-year survivors and the tumours from deceased patients with *p*-values lower than 0.01.

### Real-time RT-PCR with TaqMan

Fourteen genes that had high overall expression were selected from the 51 gene list and the 94 gene list of significantly differentially expressed genes, and scrutinized using Real-time RT-PCR with Taqman (table 3). Another two genes with homogenous expression throughout the expression array analysis were used for normalisation (table 3). All tumours except four were used, due to the lack of access to material. For each tumour, cDNA was synthesized from 1 μg total RNA (from the same RNA extraction as in the microarray experiment) using SuperScript™ III First-Strand Synthesis SuperMix for qRT-PCR (Invitrogen) according to the manufacturer's protocol.

**Table 3:** The genes used in Real-time RT-PCR.

**Genes from the 51 gene list**		
**Gene Symbol**	**Acc. number**	**validated**
CCNB1IP1	NM_182849	yes
E2F2	NM_004091	yes
GGH	NM_003878	yes
GIT2	NM_014776	yes
SERPINB9	NM_004155	yes
TMEM63A	NM_014698	yes
ZNF497	NM_198458	no
ZNF691	NM_015911	no
		
**Genes from the 94 gene list**		
**Gene Symbol**	**Acc. number**	**validated**

AKR1B1	NM_001628	yes
EGLN1	NM_022051	yes
HAX1	NM_006118	no
LGMN	NM_005606	yes
SHC1	NM_003029	no
TFAP2A	NM_003220	yes
		
**Reference genes**		
**Gene Symbol**	**Acc. number**	

PPIA	NM_203431	
PTER	NM_001001484	

Eight genes from the list of 51 genes (*p *< 0.001 and 80% presence) were selected together with six genes from the list with 94 genes (*p *< 0.01 and 100% presence), where p-values were below the significance level for the difference in average expression between 10 year survivors and deceased patients. Two genes were selected as reference genes since they had homogenous expression throughout the expression array analysis. The table also shows in which genes differences in expression between the two survival groups were validated using RT-PCR.

Real-time RT-PCR was performed in 384-well plates using the ABI PRISM^® ^7900HT Sequence Detection System (Applied Biosystems, Foster City, USA). Commercially available validated TaqMan^® ^Gene Expression Assays (including cDNA-specific primers and probes) were obtained from Applied Biosystems [15]. A keyword search for each gene name or accession number was performed, and the corresponding inventoried assay kit (500 reactions) was ordered from the website.

The PCR set up was performed using the pipetting robot Biomek FX (Beckman Coulter, Bromma, Sweden). Amplification reactions (10 μl) were carried out in triplicates with 2 μl of 1:7 diluted cDNA template, 1 × TaqMan Universal PCR Master Mix (Applied Biosystems), 1 × FAM-labelled TaqMan^® ^Gene Expression Assays Mix (Applied Biosystems) in the 384 well format. Thermal cycling was performed using the 7900HT Sequence Detection System (Applied Biosystems) with an initiation step at 95°C for 10 minutes, followed by 40 cycles of 15 seconds at 95°C and 1 minute at 60°C. In each assay, a 2-fold dilution series of five samples was recorded, and one no-template control was included.

Quantification was performed by the standard-curve method. In summary, a standard curve was recorded in each PCR assay for all genes using serial dilutions (1:2, 1:4, 1:8, 1:16, 1:32) of calibrator cDNA (sample 7011). The mean CT-value for triplicates was calculated, and the relative gene concentration of test samples was interpolated, based on the standard curve from the gene in question. All samples were normalised to the geometric mean of two endogenous controls; i.e. *PPIA *and *PTER*. The results were evaluated by testing the difference between the two groups using a one-sided Student's t-test.

## Results

### A list of 51 genes was used to cluster the tumours

**Figure 1:**
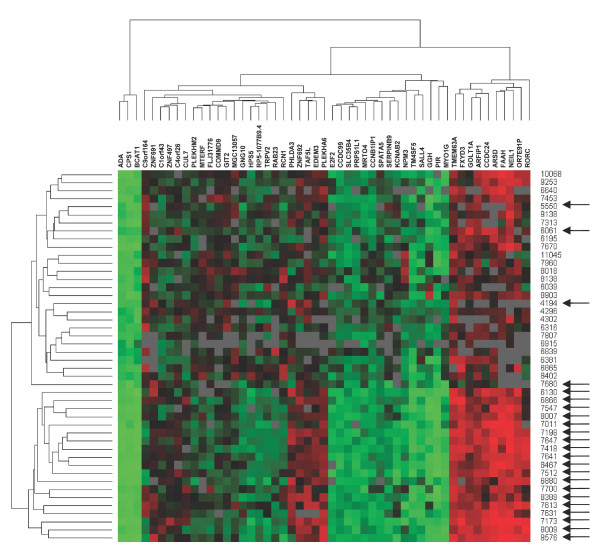
**Hierarchical clustering of the 46 tumours and 51 genes using Euclidian distance and average linkage (UPGMA).** Green represents negative values compared to the reference, red represents positive values, black represents 0, and grey represents missing values. Arrows indicate samples from patients that survived more than 10 years after diagnosis. Patients separated into two distinct clusters, one containing all deceased, together with only four survivors. The other cluster contained only tumours from survivors. Genes separated into four main clusters, characterized by distinct patterns of up- or down-regulation.

Forty-six tumours were analysed by expression microarray in order to identify genes whose expression could predict clinical outcome in node-negative breast cancer. The gene expression in 23 tumours from 10-year survivors was compared to the gene expression in 23 tumours from patients that died within ten years from diagnosis. The data generated from the microarray study was analysed using t-test analysis, resulting in a set of 51 genes that differed in average expression between the two groups of tumours with *p *< 0.001 (table 2). This gene set was used for hierarchical agglomerative clustering of the tumours using Euclidian distance and average linkage (UPGMA), resulting in two distinct clusters of tumours; one consisting of only tumours from 10-year survivors (19 tumours) and the second consisting of all 23 tumours from deceased patients and four tumours from 10-year survivors. Furthermore, the genes separated into four main clusters, and these clusters were characterized by patterns of up- or down-regulation (figure 1).

### Classification of the tumours using the gene list

**Figure 2:**
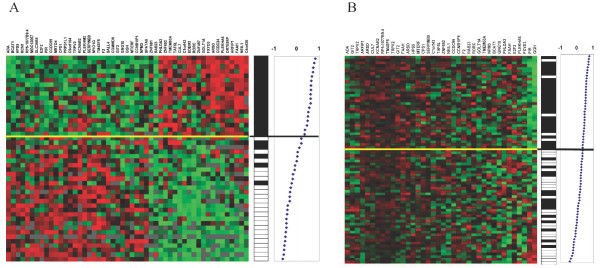
**Correlation-based classification using the 51 gene list.** A, Classification of our tumours using our 51 genes. This shows 89% accuracy and 100% NPV. B, Classification of van't Veer's tumours using our 51 genes shows 74% accuracy and 85% NPV. In A black bars represent 10-year survivors while white bars represent patients that died within ten years from diagnosis. In B, black bars represent patients that were metastasis free for five years, while white bars represent patients that developed metastasis within five years. Plots to the right show the correlation between each tumour's expression profile and the good prognosis profile.

In order to evaluate if the list of 51 significant genes could classify node-negative breast tumours into 10-year survivors and deceased patients, a correlation-based classifier using the same method as van't Veer *et al*. [3] were tested. It resulted in an accuracy of 89% and 100% NPV in our material (figure 2a).

Furthermore, the 43 classification methods available in the Weka software was tested (additional data file 2. Classification results for our own data). All classification methods were tested using leave-one-out cross-validation, which repeatedly splits the samples into a test- and training set. The best-performing classifier, VFI, only misclassified two samples in the cross-validation. In general, non-symbolic methods which use values from all genes for the classification performed better (average accuracy 85.4%, ± 4.8, 95% confidence interval) than symbolic methods which attempt to single out a subset of genes on which to base the classification (64.5%, ± 8.9). This result indicates that all (or most) of the 51 genes contribute to the survival/non-survival outcome and that a prediction of the outcome can not be based only on a small subset of these genes.

### Classification of other tumour sets using the 51 gene list

When we searched for our 51 genes in van't Veer's dataset [3], 34 of them were found and used for classification (four of them were found twice since they were represented by two replicates) (table 2). Their patients were divided into three groups; patients that were free of disease for five years or more (44 patients), patients that developed metastases within five years (32 patients), and patients with *BRCA *mutations (18 patients). The patients with *BRCA *mutations were not included in our classification. The correlation-based classifier showed 74% accuracy (figure 2b). Furthermore, the training and testing procedure, using leave-one-out cross validation, was repeated using the 34 genes resulting in an accuracy of 67% with the VFI classifier. Four of our genes differed in average expression with *p *< 0.05 between the metastasis patients and the metastasis free patients (*GGH*, *PIR*, *TAF5L *and *FAAH*).

In Wang's data 23 of our 51 genes were found (six of them were found as more than one replicate) (table 2). No specific pattern was seen and the patients that developed metastasis within five years did not separate from the metastasis free patients. In this data set three genes differed in average expression with *p *< 0.05 between the metastasis patients and the metastasis free patients (*RORC*, *FAAH *and *MRTO4*).

### Classification of our tumours using other gene-lists

**Figure 3:**
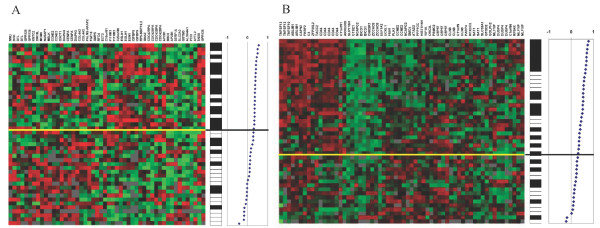
**Correlation-based classification of our tumours using other gene lists.** A, Classification of our tumours using van't Veer's genes, 72% accuracy and 73% NPV. B, Classification of our tumours using Wang's genes, 61% accuracy and 59% NPV. Black bars represent 10-year survivors while white bars represent patients that died within ten years from diagnosis.

Among the 70 significant genes identified in the study by van't Veer, 46 genes were identified in our data (additional data file 3; Van't Veer's genes in our material), of which 17 were found as more than one replicate. A VFI classification of our data using these genes showed 67% accuracy and correlation-based classification showed 72% accuracy. Six deceased patients were classified in the "good prognosis group" (figure 3a). Among Wang's 76 genes, 49 were found in our data set and 17 were found more than once (additional data file 4; Wang's genes in our material.). The VFI classifier showed 70% accuracy with these genes and the correlation-based classifier showed 61% accuracy and 59% NPV (figure 3b).

### Results of the real-time RT-PCR

Differences in gene expression between the groups were verified using Real-time RT-PCR in ten out of fourteen genes (table 3). The failure in verifying all genes might be due to sub optimal RNA quality.

## Discussion

Expression microarray analysis was performed on 46 lymph-node negative breast cancer tumours divided into two equally sized groups, where 23 tumours were from 10-year survivors and 23 tumours were from patients that died within 10 years after diagnosis. Previously, 43 of these tumours were included in a CGH study (Comparative Genomic Hybridisation) that searched for chromosomal alterations differing between deceased patients and 10-year survivors [16]. The aim of this study was to search for specific genes differentially expressed between the two groups and test the prognostic potential of these genes on other tumour sets. Previous studies have used expression microarrays to find sets of genes whose expression could predict clinical outcome in breast cancer or candidate genes that could be correlated to prognostic features [3,4,17-32]. Some of these studies focused on node-negative tumours [3,4,32]. We wanted to further expand the current knowledge of the genetic events associated with clinical outcome in lymph-node negative breast cancer using tumours from a relatively homogenous population, and compare our results with previous findings, particularly focusing on the studies reported by van't Veer *et al*. and Wang *et al*. [3,4], since both these studies developed gene-lists superior to current methods in classifying node-negative breast tumours.

**Figure 4:**
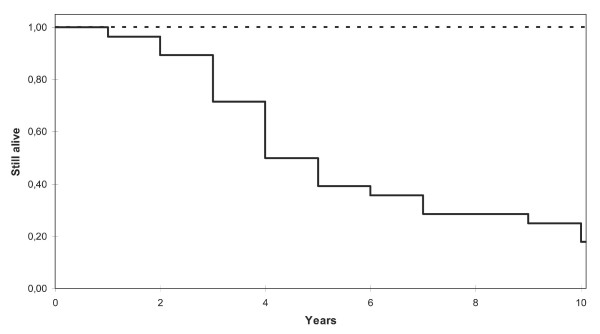
**Kaplan-Meier survival curves over time for the patients included in this study.** The correlation-based classifier classified the tumours into a "good prognosis group" and a "bad prognosis group". These Kaplan-Meier curves visualize the survival rate in the two groups the first ten years after diagnosis. The dashed line represents the good prognosis group and the black line represents the bad prognosis group.

A reporter list consisting of 51 genes with significant differential expression between the two groups (*p *< 0.001) was assembled. Hierarchical clustering using these genes resulted in two clusters, separating the two survival groups with only four survivor tumours misclassified (figure 1). Only two tumours were incorrectly classified in the cross-validation using the VFI classification algorithm (accuracy rate: 96%). The correlation-based classifier showed 89% accuracy and 100% NPV (figure 2a). We considered the NPV to be particularly important as it reflects the patient's probability to survive ten years after diagnosis if it is classified into the "good prognosis group". No patient in the "good prognosis group" died within ten years from diagnosis, which is visualized in the Kaplan-Meier curves (figure 4). Among the 18 patients that were classified in the "good prognosis group", which would not benefit from further treatment, 13 had been post-surgically treated by radio therapy, chemo therapy, hormonal treatment or a combinatory treatment (additional data file 1. Clinical data of each patient.). On the other hand, in the "bad prognosis group" 16 of the patients did not receive post-surgical treatment which they should have according to this study (of these, three were incorrectly classified 10 year survivors). Consequently, based on the results generated from the classifier using the 51 genes, the adjuvant treatment among many patients in this particular material would be reconsidered. Furthermore, the tumours within each group show similar patterns of gene expression in the 51 selected genes and these specific genes are relevant for predicting clinical outcome in our tumour material.

In order to test this gene-list further, we analysed its predictive potential in two independent data sets [3,4]. Van't Veer *et al*. identified a set of 70 genes to classify 78 node-negative breast tumours from young women into poor and good prognostic groups with an accuracy of 83% (81% when the classification threshold were calibrated so that less than 10% of the metastasis patients were classified in the "good prognosis group") [3]. Another study by van de Vijver *et al*. confirmed the relevance of the 70-gene classifier on 295 tumours, of which 151 were from node-negative patients [33]. This classifier was designed to not classify tumours from deceased patients into the "good prognosis" group, and few of the incorrectly classified tumours are from patients where the disease recurred in both studies. Recently, a mini-microarray customised based on this 70-gene classifier was tested on 162 of the lymph node-negative patients used in the previous two studies with good prognostic correlation [34]. In another study of node-negative breast cancer by Wang *et al*., a 76-gene signature that correlated to disease-free 5-year survival was developed using samples from 115 patients [4]. This set of genes showed an accuracy of 63% (93% sensitivity and 48% NPV) when tested on 171 additional tumours. These two main studies (van't Veer *et al*. and Wang *et al*.) have focused on detecting patients with good prognosis where adjuvant chemotherapy is not required. Interestingly, none of the genes used in these two studies were found in our gene list, even though some genes seems to be involved in the same pathways. Ein-Dor *et al*. suggest that since many genes are similarly correlated to breast cancer survival, several lists of genes from the same data-set would be equally predictive [35]. These lists could be rather trustworthy prognostic tools, but the specific genes in the lists are not necessarily of importance for survival if considered individually.

When classifying van't Veer's tumours based on our gene-list, the correlation-based classifier showed 74% accuracy and 85% NPV. The corresponding percentage of correctly classified tumours in van't Veer's study was 83% (88% NPV). Overall, the average time of disease-free survival of the five patients that were misclassified into the "good prognosis group" was noticeably higher than for the patients that were correctly classified into the "bad prognosis group", although the difference fell short of being statistically significant (41 months versus 28 months, *p *= 0,11). Moreover, 74% accuracy, especially with high NPV, is a good result (figure 2b). Using the VFI method, 67% of the tumours were classified correctly. The results would probably have been even better if all genes in the gene list could have been found in the material. The attempt to classify Wang's data using our genes did not provide a good correlation. This may be explained by the fact that only 23 of our 51 genes could be found in their material. Furthermore, not even Wang *et al*. themselves found particularly high accuracy when classifying their tumours (63%). Still they could specify a sub-group of 56 patients where 93% were free of metastasis within five years from diagnosis, which we could not do with our gene set. In our gene set, four genes were of special interest since they differed significantly in expression between the groups with (*p *< 0.05) in van't Veer's data as well; *GGH*, *PIR*, *TAF5L *and *FAAH*. Of these, only *FAAH *was significant in Wang's data. This gene has earlier only been correlated to multiple drug addiction [36]. In summary, our gene set had high accuracy when classifying our own material and was relatively competent in classifying the samples in van't Veer's study, but did not show high accuracy on Wang's tumours.

A correlation-based classifier showed 72% accuracy in predicting 10-year survival (figure 3a) and the VFI classification of our tumours using van't Veer's genes showed 67% accuracy. Hereby, the results both with the VFI classifier and the correlation-based classifier were moderate, considering that the NPV was only 73%. Using Wang's genes, the VFI classifier showed 70% accuracy which is moderate, while the correlation-based classifier worked poorly, showing only 61% accuracy and as many as twelve deceased patients were classified in the "good prognosis group" (figure 3b). In general, classifying a material is naturally more accurate when using a set of genes selected for that specific material. Moreover, the genes in our list seem to be more accurate in predicting 10-year survival, whereas van't Veer's and Wang's genes are likely to better predict metastasis within five years.

Many of the 51 genes with significantly different expression between the two survival groups have previously been implicated in cancer. The genes *BCAT1*, *GGH *and *SERPINB9 *have been correlated to clinical outcome in other types of cancer, such as colorectal cancer, neuroendocrine cancer, large cell lymphoma and melanoma [37-40]. The genes *SALL4 *and *TM4SF5 *were expressed to a higher extent in the tumours from deceased patients in our material and have been reported to be up-regulated in other cancers, and may thereby represent putative oncogenes [41,42]. *NEIL1*, a gene involved in DNA repair [43], showed lower expression levels in the tumours from deceased patients in our material and has been reported as down-regulated in gastric cancer [44]. The *GIT2 *gene was higher expressed in the tumours from deceased patents and may be implicated in the transformation of epithelial cells to cancer cells as well as inducing cell motility and invasion [45]. The genes *CCNB1IP1*, *CUL7 *and *E2F2 *are involved in cell cycle control and cell growth [46-48] and have expression levels in our study that promotes cell growth in the tumours from deceased patients. In our previous CGH-study of the same tumours, seven chromosomal regions were altered significantly more in the tumours from deceased patients than the tumours from 10-year survivors (4q, 5q, 6q, 12q, 17p, 18p and Xq) [16]. Five of the selected genes in the microarray study were located in these regions (*C4orf26*, *CCDC99*, *SPATA5*, *TM4SF5 *and *TRPV2*) and might be of special interest since they revealed significance in both studies.

There are disadvantages using this type of selected material for gene expression studies, the tumours in this study come from a relatively homogenous population in Sweden which might make the results less applicable for breast cancer tumours in general. Furthermore, the number of tumours investigated in this study is low, further studies using larger sets of tumours are needed to verify the significance of the 51 gene list. Also, the tumours have been frozen for a long period of time which might affect the quality of the RNA yielding less reliable results.

## Conclusion

It can be concluded that the list of 51 genes we identified (table 2) could predict clinical outcome in our material with great certainty. They were competent in van't Veer's as well but not in Wang's material, probably due to the low number of genes found in the material. In classifying our material, our gene set clearly worked best, but the genes found by van't Veer *et al*. and Wang *et al*. had some prognostic potential as well. The gene set found by Wang *et al*. had the lowest impact on our material, particularly considering the low NPV (59%) in the correlation based classification. Overall, our gene set worked similarly well in classifying van't Veer's material as their gene set on our material, slightly better considering the NPV.

The list of 51 genes might contain specific genes interesting for clinical outcome in breast cancer as well as being a good prognostic gene set. Additional studies using larger sets of tumours are needed to define the significance of these genes during the genesis of lymph-node-negative breast tumours.

## Abbreviations

BASE: BioArray Software Environment; BLAST: Basic Local Alignment and Search Tool; cDNA: complementary DNA; CGH: Comparative Genomic Hybridisation; CT: threshold cycle; Cy3: Cyanine 3; Cy5: Cyanine 5; dCTP: 2'-deoxycytidine 5'-triphosphate; DNA: deoxyribonucleic acid; FDR: false discovery rate; NPV: Negative Predictive Value; PCR: Polymerase Chain Reaction; RIN: RNA Integrity Number; RNA: ribonucleic acid; RT: Reverse Transcriptase; UPGMA: Unweighted Pair Group Method with Arithmetic mean; VFI: Voting Features Interval.

## Competing interests

The authors declare that they have no competing interests.

## Authors' contributions

EK and UD performed and analysed the microarray analysis. BO performed the statistical analysis. UD and AD performed the real time RT-PCR analysis. EK and FA evaluated the results of the rela time RT-PCR. PK provided the clinical information. EK, BO and KH interpreted the results and wrote the paper. All authors read and approved the final manuscript. KH was responsible for supervision as well as providing the funding.

## Pre-publication history

The pre-publication history for this paper can be accessed here:



## Supplementary Material

Additional file 1 Clinical data of each patient. Information about date of diagnosis, age at diagnosis, tumour size, hormone receptor status, S phase, ploidy, histology, surgery, post-surgical treatment, and survival data of each individual patient used in the study. HER2 testing was not available at the time. Click here for file

Additional file 2 Classification results for our own data. Cross-validation results for 43 classification methods available in the Weka software. Each method was evaluated using leave-one-out cross-validation (46 testing and training cycles per method, with one sample left out for testing in each run). In general, non-symbolic methods which use values from all genes for the classification performed better than symbolic methods which attempt to single out a subset of genes on which to base the classification. The best-performing classifier VFI (Voting Features Interval) only misclassified two samples in the cross-validation. Click here for file

Additional file 3 Van't Veer's genes in our material. Gene symbol and accession number for the genes in van't Veer's gene list found in our data. Click here for file

Additional file 4 Wang's genes in our material. Gene symbol and accession number for the genes in Wang's gene list found in our data. Click here for file
